# The Epigenetic Regulation in Plant Specialized Metabolism: DNA Methylation Limits Paclitaxel *in vitro* Biotechnological Production

**DOI:** 10.3389/fpls.2022.899444

**Published:** 2022-07-08

**Authors:** Ainoa Escrich, Rosa M. Cusido, Mercedes Bonfill, Javier Palazon, Raul Sanchez-Muñoz, Elisabeth Moyano

**Affiliations:** ^1^Department of Medicine and Life Sciences, Universitat Pompeu Fabra, Barcelona, Spain; ^2^Department of Biology, Healthcare and the Environment, Faculty of Pharmacy and Food Science, Universitat de Barcelona, Barcelona, Spain; ^3^Laboratory of Functional Plant Biology, Department of Biology, Ghent University, Ghent, Belgium

**Keywords:** DNA methylation, taxane biosynthesis, epigenetic regulation, Taxol, *cis*-elements, promotors, paclitaxel

## Abstract

Environmental conditions are key factors in the modulation of the epigenetic mechanisms regulating gene expression in plants. Specifically, the maintenance of cell cultures in optimal *in vitro* conditions alters methylation patterns and, consequently, their genetic transcription and metabolism. Paclitaxel production in *Taxus x media* cell cultures is reduced during its maintenance in *in vitro* conditions, compromising the biotechnological production of this valuable anticancer agent. To understand how DNA methylation influences taxane production, the promoters of three genes (*GGPPS*, *TXS*, and *DBTNBT*) involved in taxane biosynthesis have been studied, comparing the methylation patterns between a new line and one of ~14 years old. Our work revealed that while the central promoter of the *GGPPS* gene is protected from cytosine methylation accumulation, *TXS* and *DBTNBT* promoters accumulate methylation at different levels. The *DBTNBT* promoter of the old line is the most affected, showing a 200 bp regulatory region where all the cytosines were methylated. This evidence the existence of specific epigenetic regulatory mechanisms affecting the last steps of the pathway, such as the *DBTNBT* promoter. Interestingly, the *GGPPS* promoter, a regulatory sequence of a non-specific taxane biosynthetic gene, was not affected by this mechanism. In addition, the relationship between the detected methylation points and the predicted transcription factor binding sites (TFBS) showed that the action of TFs would be compromised in the old line, giving a further explanation for the production reduction in *in vitro* cell cultures. This knowledge could help in designing novel strategies to enhance the biotechnological production of taxanes over time.

## Introduction

As sessile organisms, plants must deal continuously with environmental fluctuations and external stressors. Therefore, they have developed a wide variety of mechanisms to ensure a strict gene expression regulation, allowing them to adapt their physiology efficiently. In the early ‘70s, the flow of biological information was suggested to follow from DNA to RNA and, finally, to protein (Crick, 1970). Years later, it was found that less than 5% of the genomic DNA encoded for proteins. The rest was composed of the so-called junk DNA, including transposable elements (TE) and highly repetitive DNA (Nowak, 1994). Nowadays, the initially considered “junk” DNA turned into the key to understanding how genes and genomes are regulated. A well-known example is gene promoters, which despite not being part of the coding sequence (CDS), contain the necessary elements to initiate and regulate the transcription process, representing a central role in gene regulation (Porto et al., 2014). In addition, a new layer of complexity has been found in gene regulation since not only the genetic sequences have been demonstrated to be relevant in gene transcription but also their epigenetic patterns.

Epigenetic marks include a vast range of regulatory events, such as chromatin structure remodeling, histone modifications, DNA methylation and small non-coding RNAs action, that do not involve changes in the DNA sequence (Springer and Schmitz, 2017). Epigenetic patterns have been believed to be transient and very dynamic. Nevertheless, some epigenetic variations have been proved to be meiotically heritable in plants without altering the DNA sequence (Kaeppler and Phillips, 1993), later defining the so-called epialleles. This distinctive mechanism supports the existence of an epigenetic memory in plant genomes, although the specific mechanisms are unexplored yet (Mutanda et al., 2021).

DNA methylation is an evolutionarily ancient covalent modification that involves the addition of a methyl group to the fifth position of the pyrimidine ring of cytosine bases to form 5-methylcytosine (5-mC; Zhang et al., 2018). Methylation of promoter regions is often associated with transcriptional repression, meaning gene silencing (Law and Jacobsen, 2010). In plant, cytosine methylation can take place in three different contexts: the symmetric CG and CHG and the asymmetric CHH (where H can correspond to A, T, or C; Karim et al., 2016). Regarding the establishment of the 5-mC patterns, two mechanisms have been identified: the *de novo* and the maintenance pathways, extensively described by He et al. (2011). The *de novo* mechanism establishes new patterns, while the maintenance mechanism conserves methylation patterns in the newly generated strands of DNA during the state of post-replicative DNA modification (Springer and Schmitz, 2017). Epigenetic patterns, and specifically DNA methylation, have been demonstrated to play a key role in controlling gene transcription to respond to environmental changes or stress (He et al., 2011; Zhang et al., 2018). These responses are linked with the production of a wide range of specialized metabolites to protect themselves from stress signals.

Natural products derived from plant specialized metabolism are highly demanded due to their effectiveness against a wide range of diseases, including taxanes to treat cancer (Changxing et al., 2020), tannins and flavonoids to treat diabetes (Mtewa et al., 2021) and flavonoids to treat hepatic alterations (Bachar et al., 2020), among others. However, both the low concentrations of specialized metabolites in their natural source and the over-harvesting of wild plants which have left several species endangered are impediments to meet this increasing demand (Georgiev et al., 2009). In this stage, plant cell cultures can be employed as biofactories to produce a great diversity of compounds, providing a promising, economical, and environmentally friendly solution. However, certain limitations still need to be overcome before their maximum potential is reached. One of these limitations is the gradual loss of specialized metabolite production during *in vitro* culture maintenance, which represents an important barrier in the development of large-scale production systems (Dougall et al., 1980; Deus-Neumann and Zenk, 1984; Beum et al., 2004). Why cell cultures lose the ability to produce high levels of specialized metabolites when cultured in optimized conditions while this is not happening in *in vivo* conditions, remains unclear.

Taxanes have been demonstrated to be a group of particular interest due to their unique anticancer mechanism of action. Among taxanes, paclitaxel, the active principle of Taxol^®^, is a phytopharmaceutical anticancer drug used to treat several types of cancer, including metastatic carcinoma of ovary and breast cancer, and tumors in lungs, skin, colon, kidneys, and prostate (Vidal-Limon et al., 2018). This natural antitumoral agent is a diterpenoid specialized metabolite that was first isolated in the late ‘60s. Paclitaxel blocks the mitosis progression and the activation of the mitosis checkpoint, leading to apoptosis or to the reversion of cancer cells to G0 phase (Mutanda et al., 2021). Its biosynthetic pathway comprises 19 enzyme-catalyzed steps, 15 of which are known, starting from the precursor geranylgeranyl diphosphate (GGPP) and leading to paclitaxel itself (Croteau et al., 2006). At the moment, some genes have been highlighted as putative flux-limiting genes, generating a highly controlled synthesis of taxanes (Onrubia et al., 2013; Lenka et al., 2015; Escrich et al., 2021). Nevertheless, the specific mechanism regulating taxanes biosynthesis is still not fully raveled, hindering the development of novel strategies to reach high yields of paclitaxel using rational approaches.

Available evidence shows that altered cytosine methylation patterns can be found in *in vitro* systems, leading to discrete phenotypic changes (Finnegan et al., 1996; Stelpflug et al., 2014; Ghosh et al., 2021). In addition, higher DNA methylation levels have been correlated with a low yield of specialized metabolites *in vitro* plant cell cultures (Tyunin et al., 2016; Sanchez-Muñoz et al., 2019). In our previous study, the transcriptomic and phytochemical profiling of the long-term *in vitro* maintained cell culture were performed (Sanchez-Muñoz et al., 2018). These results show that the taxane production decreases 4 times under elicited conditions. Accordingly, the genes of the taxane biosynthetic pathway present a reduced transcription activity, even in the presence of the elicitor MeJA. The longer the cell cultures had been maintained in optimized *in vitro* conditions, the greater was the number of methylated cytosines found throughout the *BAPT* promoter.

The production of certain compounds, in this case taxanes, is mainly regulated by the transcription process, and is principally controlled by the promoter structure. In plants, the transcription process is triggered by the initiation complex, recruiting the RNA polymerase near the transcription start site (TSS). Plant promoters can be structured in three regions; the core promoter, situated from −35 to +35 of the TSS (Porto et al., 2014), and the proximal and distal promoter regions, situated further from the TSS, from 200 to −100 bp and up to thousands of bp from the TSS, respectively (Peremarti et al., 2010). These regions usually contain *cis* elements that can act as enhancers or silencers by the binding of specific TFs and, specifically in the core promoter, both transcription initiators [namely the TATA-box, the Initiation Region (INR) and the Downstream Promoter Elements (DPE)] and facilitators (such as the CAAT-box and the Y-patch region) can be found. Some studies focused on transcription factor binding sites (TFBSs) demonstrated that different MeJA-responsive motifs were located in all key genes of the taxane biosynthetic pathway (Lenka et al., 2015; Yanfang et al., 2018; Zhou et al., 2019). The relation between TFBSs and gene accessibility to the RNA transcription machinery could be modified and influenced by DNA methylation, affecting gene expression and, therefore, various cellular processes (Law and Jacobsen, 2010).

The aim of this study is to provide new insights into the gradual loss of taxane production in *Taxus* spp. cell cultures maintained in optimized *in vitro* conditions and the specific methylation mechanisms involved. Therefore, a low producer *Taxus x media* cell line (of ~14 years old) and a new cell culture recently induced from fresh plant material were compared to reveal specific methylation changes during its maintenance in optimal conditions. The promoter region of three taxane biosynthetic genes was studied: the geranylgeranyl diphosphate synthase (*GGPPS*), the first gene of the diterpene pathway and, therefore, a non-specific taxane gene; the taxadiene synthase (*TXS*) gene, involved in the first step of the taxane biosynthesis; and the 3′-N-debenzoyl-2′-deoxytaxol-N-bezoyltransferase (*DBTNBT*) gene, the last gene of the pathway. With this specific set of genes, we aim to distinguish methylation patterns between three differential steps in taxane biosynthesis. The bisulfite sequencing technique was used to find different methylation patterns in these three promoters between the two age-different cell cultures, since it allows the study of methylation patterns genome-wide or in specific sequences (Cokus et al., 2008). The methylation level was determined at a single-cytosine resolution to find specific changes in the regulation of the different parts of taxane biosynthesis, as well as hotspots of DNA methylation in the three studied promoter regions. This analysis differentiates between the three possible cytosine contexts: CG, CHG, and CHH (being H any nucleotide except G). Furthermore, *in silico* analysis were carried out to identify cis-acting regulatory element involved in the MeJA-responsiveness. The integration of both processes could provide novel insights into taxane regulation, clarifying the specific mechanism influencing paclitaxel production.

## Materials and Methods

### Plant Material

*Taxus × media* callus tissue was obtained by dedifferentiation of *Taxus × media* young stem fragments (Expósito et al., 2009) and maintained for more than 10 years by biweekly subcultures on solid Gamborg’s B5 callus growing media (Gamborg et al., 1968) supplemented with 0.5% sucrose, 0.5% fructose, growth regulators [picloram (2 mg L^−1^), kinetin (0.1 mg L^−1^), and gibberellic acid (0.5 mg L^−1^)], and 1 ml of an antioxidant cocktail containing L-glutamine (29.23 g L^−1^), L-ascorbic acid (5.02 g L^−1^), and citric acid (4.99 g L^−1^). To obtain new callus cultures, *T. × media* sterilized explants were dedifferentiated on Gamborg’s B5 solid media (Gamborg et al., 1968) supplemented with 3% sucrose, growth regulators [2.4-dichlorophenoxyacetic acid (4 mg L^−1^), kinetin (1 mg L^−1^), and gibberellic acid (0.5 mg L^−1^)], and 1 ml of the antioxidant cocktail. The new callus tissue was maintained as above. In order to differentiate both calli tissues, the approximately 14-year-old callus tissue was called the old cell line (old line) and the freshly obtained was called the new cell line (new line).

### Direct Bisulfite-Sequencing

The bisulfite (BS) method has been used for detecting changes in the genome-wide methylation patterns or specific sequence context in tissue culture plants. BS treatment implies the treatment of genomic DNA with sodium bisulfite which causes cytosine conversion to uracil, while methylated cytosine (5-mC) is not converted to uracil (Cokus et al., 2008). Dellaporta protocol for plant DNA extraction was adapted to extract genomic DNA (gDNA) from 400 mg of *T. × media* callus tissue (Dellaporta et al., 1983). One μg of DNA was treated using the EpiTect Bisulfite Kit (Qiagen, Hilden, Germany). Demethylated DNA by PCR amplification was used as a control to check the conversion efficiency of treated DNA. Specific degenerated primers (Supplementary Material 1), containing Y or R instead of C or G in the forward and the reverse primers, respectively, were used to amplify overlapping segments of 150–300 bp of the promotor and the early CDS regions of *GGPPS*, *TXS*, and *DBTNBT* genes, covering from the core promoter to the starting region of the CDS. 0.5% agarose gel was used to visualize the amplified DNA fragments and purify them with the Wizard^®^ SV Gel and PCR Clean-Up System (Promega, Madison, WI, United States). Amplified and cleaned-up samples were sequenced using an ABI3730 DNA analyzer (Applied Biosystems^™^, ThermoFisher Scientific, Waltham, MS, United States).

### Analysis of Position-Specific DNA Methylation

From each sequenced sample, two Ab1 files as sequencing output were obtained, one for the forward and the other for the reverse strand. The chromatograms contained in the Ab1 files were used to determine the percentage of methylation for single-cytosine in the sequences described by Parrish et al. (2012). The relationship of T and C signals and G and A signals represented the percentage of methylation of each cytosine (mC) calculated with the formula: mC = C/(C + T) × 100, for sequences obtained using forward primers, and mC = G/(G + A) × 100, for reverse primers. With this data, we differentiate between general and specific methylation levels. For the general methylation state, we establish a cytosine as methylated when its specific methylation value is more than 50% in the analyzed sample. On the other hand, the specific cytosine methylation percentage is directly obtained from the processed Ab1 file and represents the average of the exact level of methylation in the complete cell culture. The sequences were aligned using Clustal Omega from EMBL-EBI tools (Li et al., 2015) and graphically represented by Cymate software (Hetzl et al., 2007). The master sequence was obtained from the NCBI database. As a negative control, the BS conversion of PCR amplified fragments (where the methylation of all the cytosines is lost) was used. *T*-test was performed using GraphPad Prism version 7 for Windows, GraphPad Software, California, United States. A *p* value of <0.05 (*), <0.005 (**), and < 0.001 (***) was assumed for significant differences.

### Analysis of Y Percentage

The percentage of pyrimidine (Y) presented in the promoter was represented using the sequence annotation plotting library of the DNA Features Viewer with a window size of 30 pb (Zulkower and Rosser, 2020). The promoter structure was annotated including the *cis* elements previously detected (see section Analysis of Y Percentage). The count of each base and the pyrimidine and purine percentages of the studied regions were obtained by Unipro Ugene software (v. 40.0; Okonechnikov et al., 2012).

### *cis*-Regulatory Element Prediction

Regulatory Sequence Analysis Tools for Plants (RSAT Plants; Medina-Rivera et al., 2015) and PlantCARE (Lescot et al., 2002) were used to predict the *cis*-regulatory elements present in the core promoter. The online tool RNA polymerase II promoters in plant DNA sequences TSSPlant (Softberry Inc.; Solovyev et al., 2010) was used to predict the transcription start sites (TSSs) and the presence of TATA-box in the selected promoters. The *cis*-regulatory elements involved in methyl-jasmonate (MeJA) and ethylene responses were predicted using PlantPAN 3.0 (Chow et al., 2019).

### Accession Numbers

The analyzed genes were obtained from the NCBI database and are freely accessible under the following accession numbers: AY566309.1 (*GGPPS* complete gene, *Taxus × media*), EF153471.1 (*TXS* promoter and partial CDS, *Taxus cuspidata*), FJ603644.1 (*DBTNBT* promoter, *T. cuspidata*), and AY563629.1 (*DBTNBT* partial promoter and complete CDS, *T. cuspidata*).

## Results

### Promoter Methylation Patterns

**Figure 1:**
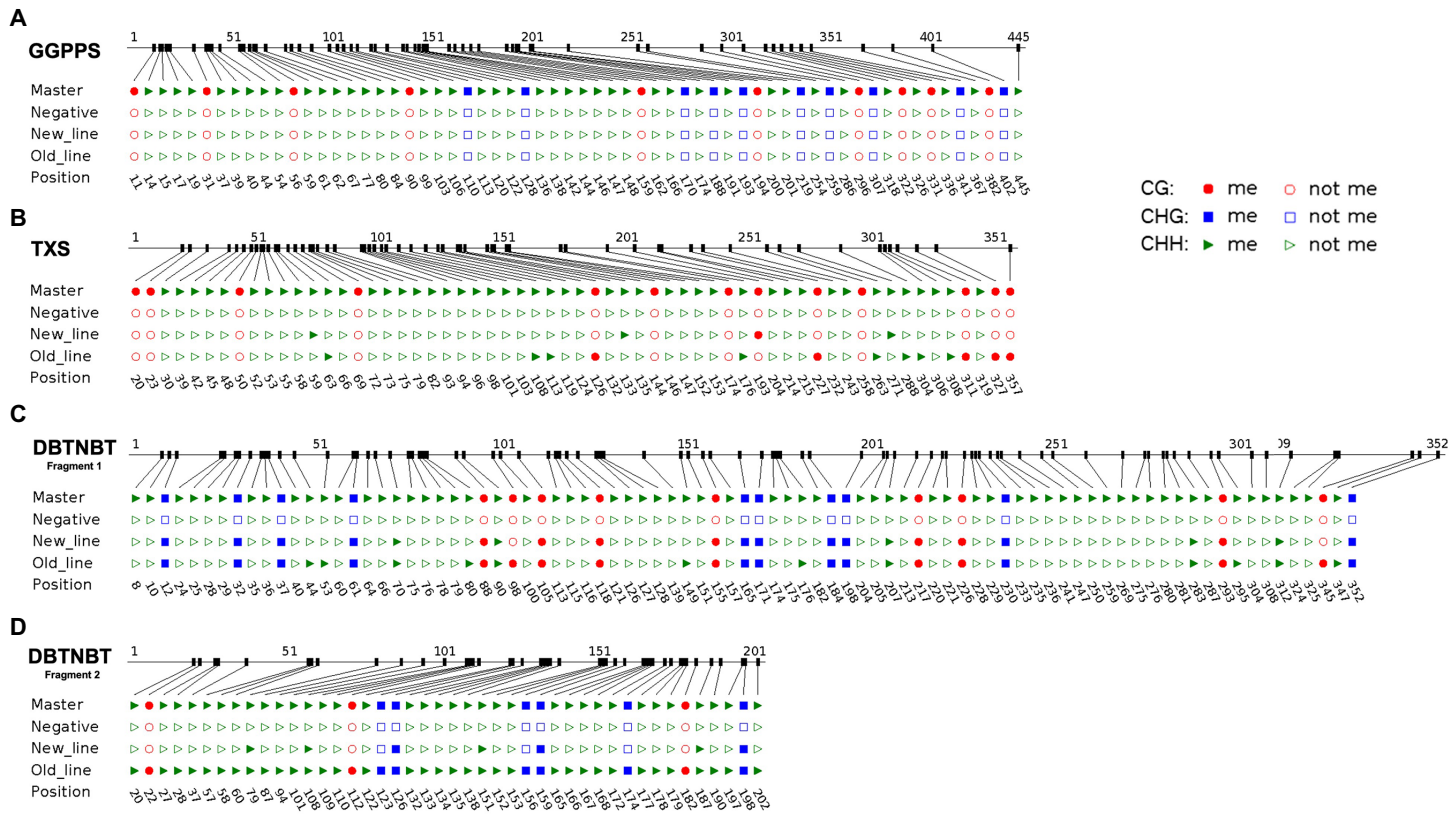
Bisulfite-sequencing methylation analysis of the partial promoter region in the old and the new *Taxus x media* cell line. **(A)** *GGPPS* partial promoter. **(B)** *TXS* partial promoter. **(C)** *DBTNBT* partial promoter 352 nucleotides before TSS, fragment 1. **(D)** *DBTNBT* partial promoter from TSS to CDS, fragment 2. Master and negative sequences correspond to the untreated and the unmethylated sequences, respectively. The three different cytosine contexts are indicated in red spheres (CG), blue squares (CHG) and green triangles (CHH), colored (methylated) or empty (not methylated).

In order to discriminate different methylation patterns through the taxane biosynthetic pathway we selected three genes involved in different points of it: *GGPPS* as a non-specific taxane biosynthetic gene and shared by other plant physiological processes, *TXS* as the first specific taxane biosynthetic gene, and *DBTNBT*, the last step of taxane biosynthesis. The general methylation state where the cytosines were represented as methylated or not was defined to detect methylation hotspots within the sequences. In Figure 1, it is represented the distribution of the methylated cytosines through the selected promoter regions in both the old and new cell line, clearly showing notable differences between the three genes.

The analyzed *GGPPS* promoter region did not present methylated cytosines in any context, neither in the new line nor the old line (Figure 1A). On the other hand, the *TXS* promoter region presented a slightly higher methylation level, presenting a differential methylation pattern comparing the new line and the old line (Figure 1B). While the new line only showed four methylated cytosines, the old line had 13 methylated cytosines, seven of them were in the 100 nucleotides upstream of the CDS. Interestingly, the study of the cytosine content in the *TXS* promoter region shows a completely different composition due to the lack of CHG context (Figure 1B).

The *DBTNBT* promoter was split up into two different representations due to the distance between the TSS and the CDS (see section Analysis of Y Percentage). Its cytosine composition and methylation pattern are represented by a region of 352 nucleotides before TSS, and a region of 202 nucleotides from the TSS to the CDS (Figures 1C,D, respectively). Methylated cytosines were found in the *DBTNBT* promoter in both the new and the old cell lines in all three contexts. The cytosine methylation pattern before the TSS showed that almost all CG and CHG cytosine contexts were methylated in both new and old cell lines with 92.31% and 96.51% of methylated cytosines, respectively. This methylation pattern was not detected in the other two genes previously studied, suggesting a differential methylation regulation focused on this specific part of the pathway.

Moreover, in the region represented before TSS, the old line showed more methylated CHH than the new line. The study of the *DBTNBT* promoter region after the TSS showed a non-homogeneous (methylated and unmethylated) cytosine distribution, showing an unequal cytosine accumulation through its sequence. In addition, the methylated cytosines were found in a specific region of approximately 180 nucleotides (Figure 1D). Furthermore, the distribution of the cytosines (methylated or not) were different from position 108 until 179, being clustered in groups of 4–5 cytosines.

Table 1 represents the study of the general state of methylation in each promoter, as well as the specific methylation levels of each cytosine for each cell line (for more details, see section Analysis of Position-Specific DNA Methylation). The *GGPPS* promoter showed unmethylated cytosines along the studied region, and, consequently, the general methylation state is zero in all three contexts (Figure 1A; Table 1). Concordantly, the specific cytosine methylation percentage was low in all three cytosine contexts for both cell lines. The *TXS* promoter region presented differences in the general and specific cytosine methylation states between both cell lines. The CG context and the total percentage of specific methylated cytosines showed a significant difference between the two cell lines, although the differences showed in the total amount could be attributed to the CG methylation context.

**Table 1:** Methylation state in studied promoter regions of *GGPPS*, *TXS*, and *DBTNBT*.

Promoter	Cell culture	General cytosine methylation (%)				Specific cytosine methylation (%)			
		CG	CHG	CHH	Total	CG	CHG	CHH	Total
GGPPS	New line	0	0	0	**0**	6.01	3.90	1.91^*^	**2.99** ^**^
	Old line	0	0	0	**0**	4.17	8.55	8.32^*^	**7.66** ^**^
TXS	New line	7.69	-	6.52	**6.67**	8.11^**^	-	10.65	**10.09** ^**^
	Old line	38.46	-	17.39	**21.67**	39.93^**^	-	17.10	**22.42** ^**^
DBTNBT	New line	71.43	68.42	10.14	**22.48**	60.01^*^	53.41^**^	13.61^***^	**24.74** ^***^
	Old line	100	100	43.75	**58.14**	92.31^*^	96.51^**^	60.08^***^	**56.78** ^***^

The general cytosine methylation level is represented as the percentage of cytosines reaching 50% methylation. The specific cytosine methylation level is represented as the average of the methylation level of each cytosine context. A *p* value of < 0.05 (^*^), <0.005 (^**^), and < 0.001 (^***^) was used for significant differences. Total percentage of the general and specific cytosine content is indicated in bold.

The analysis of the specific methylation state in the *DBTNBT* promoter revealed that the three cytosine contexts presented significant differences, although the CHH context presented the most significant differences (Table 1). On the *DBTNBT* fragment from the TSS to CDS, the new line showed few methylated cytosines compared with the old line, which showed a significant highly methylated cytosine region whose mainly correspond to CHH cytosines (Figures 1C,D). Surprisingly, in the *DBTNBT* promoter region, before the TSS, both lines had at least more than 90% of CG and CHG methylated cytosines (Table 1; Figure 1C). However, in the region from the TSS to the CDS no CG methylated cytosines were found, and less than 50% of the CHG cytosines were methylated in the new line (Figure 1D). Interestingly, these results highlighted that the differential epigenetic regulation through the *DBTNBT* promoter region, as well as that the significant difference in the total amount of specific methylation could be mainly attributed, as expected, to the changes in the CHH context (Table 1).

### Pyrimidine Percentage and *cis*-Regulatory Element Prediction

**Figure 2:**
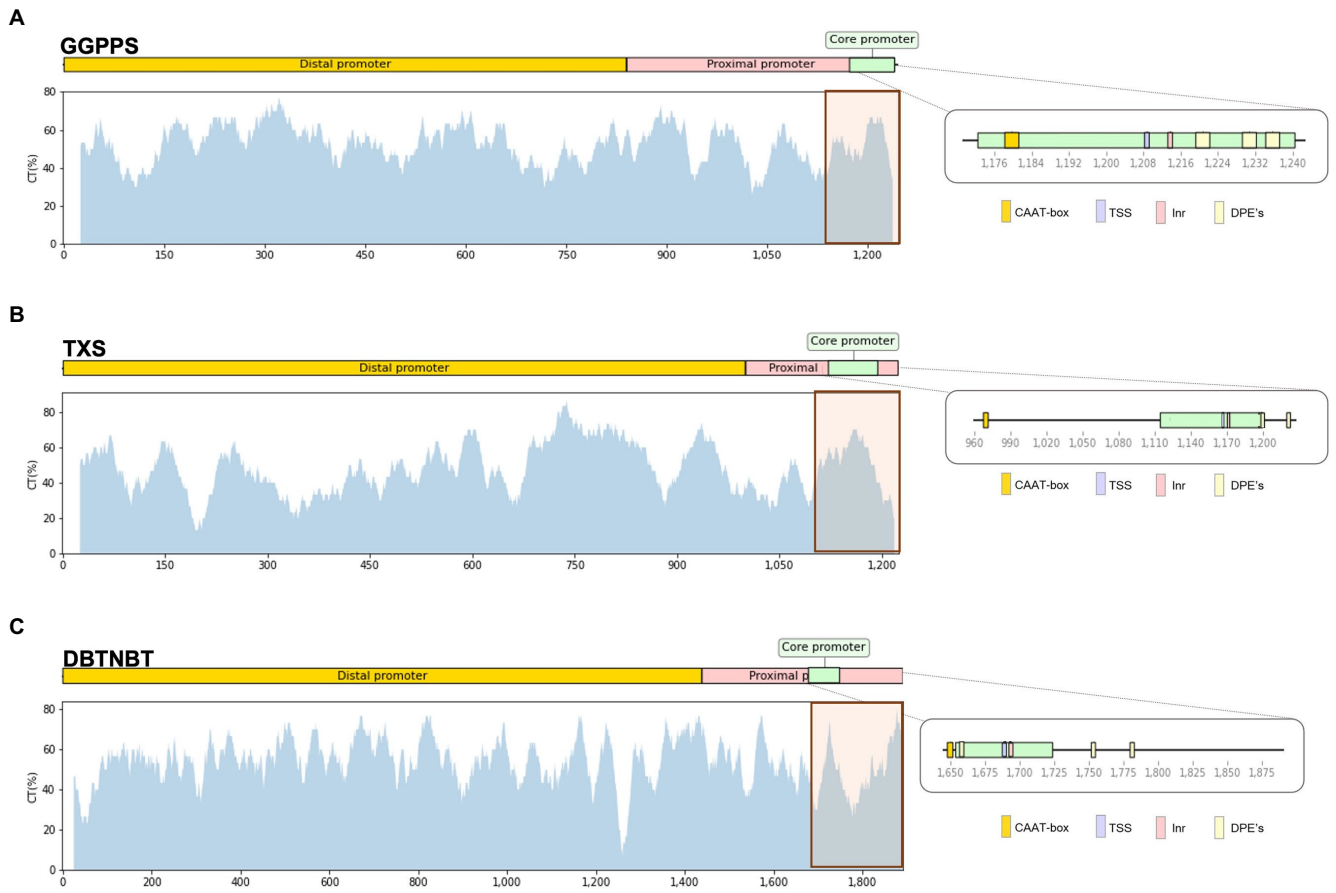
Percentage of pyrimidine presence in the promoter. It is represented the distal, proximal, and core promoter. The predicted cis elements are detailed in the zoomed core promoter. **(A)** *GGPPS* promoter, **(B)** *TXS* promoter and **(C)** *DBTNBT* promoter.

The heterogeneous distribution of cytosines through the selected promoters motivated the further study of the pyrimidine composition of these regions. Pyrimidine (Y) accumulation is often related to the presence of a Y-patch region, usually located in the proximal promoter between the TSS and the CDS (Yamamoto et al., 2007a). In addition, a prediction of *cis*-regulatory elements was performed to identify possible targets of the Y accumulation. As the first result of this approach, the three genes studied showed to be TATA-less promoters (Figure 2). Therefore, they required of other *cis* elements to finely regulate gene transcription. As expected, the three promoters showed the minimal *cis*-motifs for transcriptional activity regulation near the TSS: the INR, several DPEs and the CAAT-box. The putative Y-patch region (orange box) is defined as a region, rich in pyrimidine content, that involves at least 100 nucleotides upstream of the CDS and comprises the core promoter and the TSS (Figure 2).

The determination of pyrimidine content of the three promoters under study (Figures 2A–C) predicted the location of the putative Y-Patch region. The selected accumulations of %Y were located from the position 1,113 to 1,240 for the *GGPPS* promoter (Figure 2A); from 1,067 to 1,221 for the *TXS* promoter (Figure 2B) and from 1,796 to 1,891 for the *DBTNBT* promoter gene (Figure 2C). These three regions were defined as Y-regions.

Table 2 shows that the three promoters, *GGPPS*, *TXS* and *DBTNBT*, present a nearly equal ratio of pyrimidines and purines, around 50%. On average, the difference of pyrimidine and purine percentage in *GGPPS* and *TXS* oscillates ±2.3 and 2%, respectively. The deep study of the nucleotide composition showed that *GGPPS* promoter composition remarkably presented only two and four cytosines in the core promoter and putative Y-region, respectively, whose are clearly underrepresented in relation with the other nucleotides (Table 2). That reduces the potential methylation sites due to the shortage of cytosines. The core promoter of *TXS* presented a variance of ±4.1% in the composition of pyrimidine and purine and, as above-mentioned, there was an accumulation reaching 66% of Y content. Nevertheless, observing the single nucleotide composition, this pyrimidine percentage is due to the quantity of thymine located in this region rather than cytosines (Table 2). As occurs with the *GGPPS* promoter, this fact significantly reduces potential methylation sites. Pyrimidine levels in the *DBTNBT* promoter present the highest rate of Y content compared to *GGPPS* and *TXS* promoters (Table 2). In this case, the prediction of *cis* elements in the *DBTNBT* promoter locates the TSS at position 1,689, showing a long distance from the TSS to the CDS (~200 bp; Figure 2C). This distance was highlighted by an orange box containing two separate pyrimidine accumulations. The valley between these two accumulations corresponds to the cytosine shortage located in positions 1 to 100 in the previous graphical representation (Figure 1D).

**Table 2:** Pyrimidine and purine percentage and single nucleotide composition of *GGPPS*, *TXS*, and *DBTNBT* promoters.

		CT (%)	AG (%)	C	T	A	G
*GGPPS*	Promoter	52.3	47.7	178	471	403	188
	Proximal promoter	49	51	27	120	78	25
	Core promoter	50.7	49.3	2	34	17	18
	Y-region	**50.9**	**49.1**	**4**	**54**	**25**	**31**
*TXS*	Promoter	48	52	197	389	434	201
	Proximal promoter	45	55	46	89	116	49
	Core promoter	54.1	41.9	8	35	24	7
	Y-region	**52.3**	**47.6**	**15**	**53**	**44**	**18**
*DBTNBT*	Promoter	54.4	45.6	458	569	466	398	
	Proximal promoter	50.5	50.5	65	84	119	32	
	Core promoter	50.7	49.3	12	27	31	7	
	Y-region	**57.3**	**42.7**	**35**	**28**	**33**	**14**

Bold numbers represent the nucleotidic composition of the putative Y-region.

The nucleotide composition of the whole *DBTNBT* promoter sequence oscillated ±4.4 from the balanced value of 50% (Table 2). However, along the proximal promoter and core promoter, the composition of purine and pyrimidine was balanced. The Y-region of the *DBTNBT* promoter presents the highest unbalanced pyrimidine–purine percentage, reaching a variance of ±7.3%. Surprisingly, in discordance with *GGPPS* and *TXS* promoters, the main pyrimidine base in this Y-region is cytosine representing more than 50%. The high rate of cytosines in this specific region of the *DBTNBT* promoter sequence indicates that this region could be highly regulated by DNA methylation mechanisms.

### Binding Site of MeJA and Ethylene Transcription Factors

**Figure 3:**
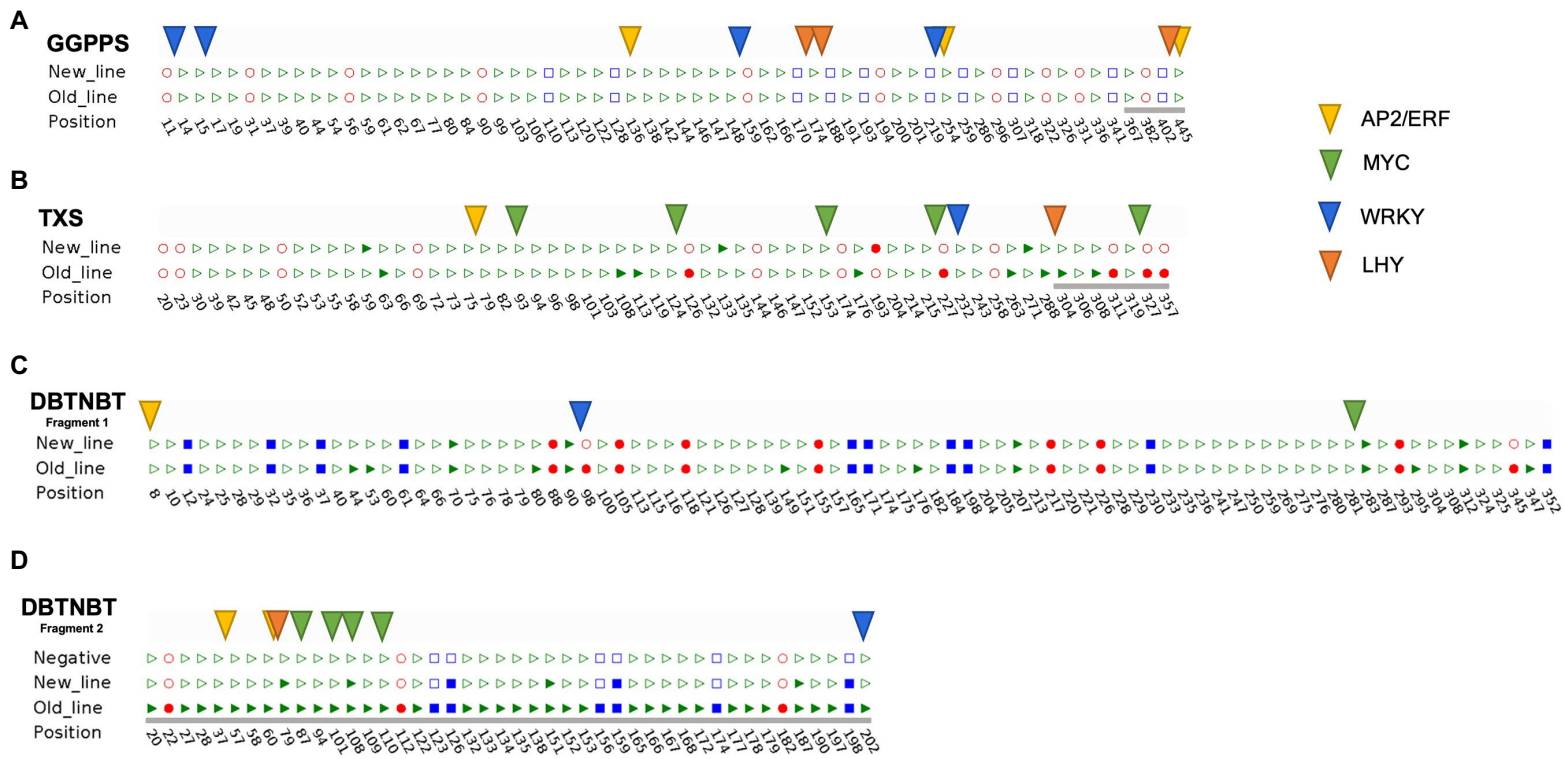
*cis*-acting regulatory elements involved in defense and stress responsiveness in the partial promoter regions of *Taxus x media* cell lines. **(A)** *GGPPS* partial promoter. **(B)** *TXS* partial promoter. **(C)** *DBTNBT* promoter region before the TSS, fragment 1. **(D)** *DBTNBT* promoter region from the TSS to the initial region of the CDS, fragment 2. The three different cytosine contexts are indicated in red spheres (CG), blue squares (CHG) and green triangles (CHH), colored (methylated) or empty (not methylated). The transcription factors studied are AP2/ERF, MYC, WRKI, and LHY. The distance between the TSS and CDS is highlighted by a gray line.

To study the role of DNA methylation in plant stress responses, *cis*-regulatory elements in response to MeJA and ethylene signals have been predicted and located through promoters due to their established relationship with plant specialized metabolism. From these data, we integrated the predicted *cis* elements and the methylation patterns found in the *GGPPS*, *TXS*, and *DBTNBT* promoters (Figure 3).

First, we found that independently of the promoter studied, the vast majority of predicted TFBSs were clustering in the proximal zone of the CDS. Despite this, a differential presence of *cis*-acting regulatory elements through the studied promoters was found. The *TXS* and *DBTNBT* promoters presented the conserved motifs, CGTCA-motifs, G-boxes, and E-boxes, all related to responsiveness to MeJA. Discordantly, the *GGPPS* promoter did not show any of these *cis* elements in its promoter sequence, excepting a low affinity E-box (Supplementary Material 2).

In order to detect binding sites for specific transcription factors, we focused on AP2/ERF, MYC, LHY, and WRKY transcription factors binding sites (TFBSs). Regarding to MYC TFBSs, the *GGPPS* promoter region did not present any of them, but *TXS* and *DBTNBT* did. The *GGPPS* promoter showed the richest sequence in WRKY TFBSs, presenting the highest number of W-boxes (Figure 3A). MeJA responses had been also related to the overexpression of ethylene-responsive transcription factors such as the AP2/ERF, a *cis*-regulatory element that can be found through all the studied promoters.

The effect of the cytosine methylation points over the predicted TFBSs show relevant information when both lines, the new and the old, were compared. First, and due to the lack of methylated cytosines in the *GGPPS* promoter region, no TFBS were related to methylation accumulations. The methylation points in the *TXS* promoter of the new line did not seem to overlap any TFs involved in the MeJA response. However, the old line methylation pattern presented methylated cytosines in the same position as LHY and MYC TFBSs (Figure 3B). Interestingly, 3 of 5 MYC TFBSs overlap with methylation hotspots in the old line *TXS* promoter. WRKY and AP2/ERF TFs, on the other hand, were not showing any associated methylation accumulation.

In the case of the *DBTNBT* promoter region, most of the predicted TFBSs are located in a region of 40 nucleotides around the start of the CDS, matching with the distribution previously observed in the previous promoters. Nevertheless, the presence of the TFBSs in the *DBTNBT* region is clustering at a specific point of the sequence. The WRKY TFBSs were not associated with methylated cytosines in the new line, but these positions were methylated in the old line. The *cis* elements related to the ethylene response transcription factors (AP2/ERFs) were affected in the new and the old line. As expected, the MYC TFBSs in the *DBTNBT* promoter shown methylation accumulation in the old line. Clearly, the distribution of *cis* elements in response to MeJA and ethylene signaling along the three promoters studied was not homogeneous, also showing a differential accumulation of methylation cytosines through their sequences. In addition, and, surprisingly, the promoter region showing a higher methylation accumulation, the *DBTNBT* promoter, also showed a cluster of TFBSs overlapping with the above-mentioned region of the methylation hotspot.

## Discussion

Methylation patterns alterations in specialized metabolism genes have been demonstrated to lead to an important reduction in the yield of plant *in vitro* cell cultures over time, as the expression of these genes can be drastically reduced. The identification of promoters accumulating high ratios of methylation can be indicative of regulatory points of a biosynthetic pathway, as well as key transcription factor binding sites. In this study, with the aim to clarify the role of the methylation mechanisms in the previously demonstrate transcriptional alterations of taxane biosynthetic genes, we selected three genes of this pathway, one of them belonging to the primary metabolism, the *GGPPS* gene, and the other two, the first (*TXS*) and the last gene (*DBTNBT*) involved specifically in the paclitaxel biosynthetic pathway. The study, using two aged-different *Taxus × media* cell cultures, could lead us to demonstrate the correlation between the methylation increment in key biosynthetic genes and the gradual loss of taxane production during its long-term maintenance in optimal *in vitro* conditions.

The methylation study performed reveals differential methylation patterns between the three promoters, being the last gene of the pathway (*DBTNBT*) the most methylated. Moreover, the accumulation of methylation points was considerable in the old line. As it was demonstrated by Sanchez-Muñoz et al. (2018), the *in vitro* maintenance of *T. x media* cultures increases the cytosine methylation in *BAPT* gene, a key gene involved in taxane production. The optimal growing conditions could cause a lack of stressing stimulus; therefore, the cell would silence the taxane biosynthetic pathway.

The deep study of each promoter reveals that the *GGPPS* promoter is the least affected by methylation mechanisms. We demonstrated that *GGPPS* promoter presented low levels of methylated cytosines and, in addition, a scarce cytosine content in the core promoter (Figure 1A; Table 2). This region correspond with the presence of the putative Y-patch (Yamamoto et al., 2007a).The unusual cytosine content prevents methylation accumulation and, consequently, avoids gene silencing. Therefore, the *GGPPS*, a non-specific taxane biosynthetic gene, is expected to not be affected by methylation, since it is a common precursor of the primary plant metabolism, such as the gibberellin biosynthetic pathway. In addition, the *T. x media* cell culture *in vitro* maintenance along the time does not alter the methylation pattern of the *GGPPS*, allowing the plant development.

In contrast, the *TXS* gene is specifically involved in the taxane biosynthetic pathway. It is observed that it is more affected by methylation mechanisms, especially in the old line. In this case, the maintenance in *in vitro* conditions increases the methylation levels of our cell cultures, as previously demonstrated in the *BAPT* promoter (Sanchez-Muñoz et al., 2018). Moreover, the highest accumulation of methylated cytosines, ~60%, was located in the putative Y-patch region. This region has a characteristic sequence, TCTCTCTTC, located from position 303 to 311 (Figure 1B), corresponding to patterns associated with the presence of Y-patch Yamamoto et al. (2007b). The methylation of these regulatory patterns in the old line indicates that the *TXS* gene is regulated by Y-patch motifs and the methylation of these motifs in the old line demonstrate that the gene expression is affected by *in vitro* culture maintenance.

In addition to the methylation mechanisms and the Y-patch motifs, other factors influence the regulation of gene expression, as well as the cytosine patterns. The studied *TXS* promoter region does not present the CHG context, reducing the methylation context possibilities. Further studies could be focused on the effect of the lack of CHG context and its relationship with the methylation mechanisms.

Surprisingly, the *DBTNBT* promoter structure differs from the other two promoters studied. On the one hand, the TSS is located almost 7 times further upstream of the CDS than the other promoters. On the other hand, there is a notable presence of cytosines in the *DBTNBT* promoter (24%) compared to the *GGPPS* and *TXS* promoters (14 and 16%, respectively), thus increasing the putative methylation points and the possible presence of Y-patch. In relation to cytosines, the three contexts are present in this promoter. The CHH context was the least methylated on the new line and showed the most significant differences between the two lines. Hence, the CHH context, only methylated by *the novo* mechanism is known to be the most dynamic one (He et al., 2011). It explains its implication in the adaptative response of cell cultures in *in vitro* conditions. Consequently, it is not surprising that the new line has very low levels of CHH-methylation relative to the old one, showing that the culture time increases the level of *de novo* methylation in *DBTNBT* promoter in *Taxus* spp., cell cultures, and therefore modifying the pathway productivity.

Regarding the possible presence of Y-patch in the *DBTNBT* promoter, this was located after the TSS with a characteristic distribution of cytosines. The study of pyrimidine composition in *DBTNBT* promoter (Figure 2) revealed that Y accumulations before the CDS belonged to the presence of cytosine (79%) instead of thymine, as opposed to the *GGPPS* and *TXS* promoters (Table 2). This analysis justifies the presence of the Y-patch region in the *DBTNBT* and its susceptibility to being affected by methylation mechanisms. Interestingly, this region of the old line was completely methylated. These facts imply that the *DBNTBT* gene is highly regulated, and its expression is affected by the time the cells are exposed to *in vitro* conditions. Therefore, these results could be linked with the low presence of paclitaxel, almost undetectable, in the taxane profiling of the old cell line (Sanchez-Muñoz et al., 2018). The high quantity of cytosines and the high methylation ratio found in the *DBTNBT* promoter, together with the results of the study of the *BAPT* promoter by Sanchez-Muñoz et al., 2018, showed that the last steps of the taxane pathway are strongly regulated.

Particularly on the CG cytosine contexts, the three promoters studied showed some non-CG methylated, especially in the new line. In the studies reviewed by Kenchanmane Raju et al. (2019), it was reported that plant defense responses are enhanced in non-CG methylated mutants, highlighting the limiting role of DNA methylation in stress responses (Luna and Ton, 2012; Slaughter et al., 2012; Kenchanmane Raju et al., 2018). The *GGPPS* promoter did not present any methylated CG. The CGs along the *TXS* promoter were only methylated in the old line. Regarding the *DBTNBT* promoter in the region after the TSS, all CGs are methylated in the old line. This could be explained by the fact that in *in vitro* conditions, plant cells progressively reduce the need for cell defense responses, and concordantly there is a progressive methylation accumulation in specific points of promoter related to specialized metabolism. The region between the TSS and the CDS in the *DBTNBT* promoter seems to have a pivotal role in taxane production regulation.

To understand how the expression of the three studied genes is regulated, the location of transcription factors is also a key aspect. The presence of *cis*-acting regulatory elements through *GGPPS*, *TXS*, and *DBTNBT* promoters was predicted in relation with MeJA and ethylene responses. Previous studies of taxane gene expression demonstrated that MeJA increases the taxane production (Cusido et al., 2014). The *TXS* and *DBTNBT* promoters present elements related to the ability to respond to MeJA, such as the CCGTCA motifs, G-box, and E-boxes, demonstrating a relevant putative regulatory role in taxane biosynthesis. The study of these *cis* elements showed that the distance between the TSS and the CDS in the *DBTNBT* promoter is longer than the other taxane biosynthetic genes studied (Figure 2C). As mentioned by Yamamoto et al. (2011), the core type affects the gene structure as well as the expression profile; the longer distance between TSS and CDS, the lower the expression ratio of the gene. This finding is also supported by the fact that the *DBTNBT* promoter is a TATA-less promoter and is differently regulated than the *BAPT* gene, a TATA-type promoter that shows a different promoter structure. The TATA-type promoter has a shorter distance between the TSS and the CDS, while in TATA-less types, this distance can be extended (Yamamoto et al., 2011). Considering that *BAPT* and *DBTNBT* are the last steps of the taxane biosynthetic pathway, it is expected that their expression would be strictly controlled.

As mentioned above, specialized metabolism is closely related to stress and defense responses, of which the best characterized are those related to MeJA and ethylene signals. Both signals have been demonstrated to influence taxane biosynthesis (Cusido et al., 2014). And the action of specific transcription factors such as MYC (Lenka et al., 2015; Cui et al., 2019), AP2/ERF (Zhang et al., 2021), LHY and WRKY (Zhou et al., 2019) has been shown to be responsible for the effects in taxane production. According to our results, the *GGPPS* promoter has the highest amount of WRKY TFBSs located along the studied region (Figure 3A). The WRKY TFs, aside from being related to the MeJA response, have also been demonstrated to be a gibberellic acid (GA_3_) repressor. This correlates with the crosslink between *GGPP*, GA_3_ and taxanes synthesis. The WRKY TFs might be an on–off switch control to regulate the primary and specialized metabolism, hence avoiding the competition between the two metabolisms for GGPP. Regarding the *TXS* and the *DBTNBT* promoter, the distribution of the *cis*-acting regulatory elements shows TFBSs with a putative altered accessibility due to the methylation of the cytosines in the old cell line (Figure 3). Contrary, in the new line, the action of TFs should not be affected by methylation in the *TXS* promoter (Figure 3B). It should be pointed out the interesting results obtained in the *DBTNBT* promoter in the old line, where almost all MYC TFBSs predicted are accumulated between the TSS and the CDS, being all of them affected by methylation accumulation (Figure 3D). This evidence is in the line with Niederhuth and Schmitz (2017), where their study in *A. thaliana* showed that the majority (72%) of TFs binding is inhibited by methylation. According to these results, altogether with the transcriptomic and production alterations showed in previous study (Sanchez-Muñoz et al., 2018), it can be concluded that the production of taxanes could be epigenetically modulated by the methylation of these TFBSs, especially in the last steps of the pathway.

After performing an exhaustive study through the promoters, new insights about taxane biosynthesis regulation regarding epigenetic mechanisms have been provided. First, the *GGPPS* promoter has the region from the TSS to the CDS protected against methylation accumulation, since its cytosine distribution may prevent gene silencing caused by 5’mC methylation. The *TXS* promoter does not present the CHG context in the region analyzed. Consequently, only two of the three cysteine contexts could be methylated, offering the possibility of a slightly regulation in this first step of the pathway. Finally, the *DBTNBT*, as the last gene of the taxane pathway, seems to be strongly regulated. This seems to be achieved by two independent mechanisms. On the one hand, by the distance between the TSS and the CDS that the structure of the promoter is showing and, on the other hand, by the high rate of cytosines presented in this region, making a hotspot of cytosines that can be potentially methylated. In addition, the distribution of *cis* elements showed that the region between the TSS and the CDS is a key transcriptional regulator in this gene. These results, provide strong evidences to confirm that the last steps of taxane biosynthesis are key for the regulation of paclitaxel production. Moreover, it proves that epigenetic mechanisms are directly involved in silencing these genes, being our results for the last gene of the pathway perfectly correlated with the results in the *BAPT* promoter shown by this author. Last but not least, our study provides convincing evidence that the long-term effects of the *in vitro* conditions differentially affect the gene regulation of plant primary and specialized metabolism.

In conclusion, epigenetic methylation has shown to be an essential player in the expression of the last steps of taxane biosynthesis, mainly due to its close relationship with their promoter structure, particularly evident in the *DBTNBT* promoter. In this study, it has been demonstrated that epigenetic control depends not only on the gene and its function but also on the *in vitro* culture maintenance time. Besides, it is shown that methylation accumulation is a specific and directed mechanism rather than a general methylation accumulation through the entire genome. Furthermore, the transcription factors and *cis* elements are strategically located along the promoter over regions easily methylated by the *de novo* mechanism. All this evidence is not only providing new knowledge about the specific mechanism under the epigenetic regulation of specialized metabolites but also novel insights that will ease the design of strategies for the rational improvement of paclitaxel production, such as the modification of *DBTNBT* or *BAPT* promotor sequences by genome editing technologies as CRISPR.

## Data Availability Statement

The original contributions presented in the study are included in the article/Supplementary Material, further inquiries can be directed to the corresponding authors.

## Author Contributions

JP, RS-M, and EM conceived the project and designed the research plan. AE performed the bisulfite method and computational work. RC and MB supervised the DNA methylation analysis. RS-M and EM supervised the computational work. AE, RS-M, and EM wrote the manuscript. MB, RC, and JP supervised and complemented the writing. All authors contributed to the article and approved the submitted version.

## Funding

This work has been carried out at the Plant Physiology Laboratory (Universitat de Barcelona). It was financially supported by the Spanish PID2020-113438RB-I00/AEI/10.13039/501100011033 and the Generalitat de Catalunya 2017SGR242. AE was supported by a fellowship from the Universitat Pompeu Fabra.

## Conflict of Interest

The authors declare that the research was conducted in the absence of any commercial or financial relationships that could be construed as a potential conflict of interest.

## Publisher’s Note

All claims expressed in this article are solely those of the authors and do not necessarily represent those of their affiliated organizations, or those of the publisher, the editors and the reviewers. Any product that may be evaluated in this article, or claim that may be made by its manufacturer, is not guaranteed or endorsed by the publisher.

## Supplementary Material

The Supplementary Material for this article can be found online at: https://www.frontiersin.org/articles/10.3389/fpls.2022.899444/full#supplementary-material
