# Supplementary material for: The Epigenetic Regulation in Plant Specialized Metabolism: DNA Methylation Limits Paclitaxel in vitro Biotechnological Production
Source: Front Plant Sci. 2022 Jul 8;13:899444. doi: 10.3389/fpls.2022.899444 (PMC9305382; doi:10.3389/fpls.2022.899444)
Supplement: Supplementary file 1 [file Table_1.DOCX]

| *Supplementary material 1.* **Degenerate primer pairs used to amplify and sequence different fragments of the GGPPS, TXS and DBTNBT promoters.** | | | | |
| --- | --- | --- | --- | --- |
| **FRAGMENT** | **Gene** | **Forward** | **Reverse** | **Sequence (5'-3')** |
| 1.1 | GGPPS | x |  | GGAGGATAAAATTTAAYAGTYAAATG |
|  | GGPPS |  | x | CAAAATTTCAAATATCCTRCTTTRCTC |
|  | GGPPS | x |  | GGTYAATGGGTGTTATATTTTAT |
| 1.2 | GGPPS | x |  | GGAAGGTGTGTGTGAAAATTTGAAG |
|  | GGPPS |  | x | CCATAATCTCACAAATTARCARCACC |
|  | GGPPS | x |  | GTGTGAAAATTTGAAGAAAATTAAYAAG |
| 1.1 | TXS | x |  | AAGTGTTYYGTATGTGAYTAAYTAGA |
|  | TXS |  | x | TTTTRARTTRARARRTATTTTCAAA |
|  | TXS | x |  | AAGYTAGYATGTTTTTYTTGGTT |
| 1.2 | TXS | x |  | ATTAYATTGYTTTGATYTAATTGYTA |
|  | TXS |  | x | TCACRTRTTTAATCARATATTCATRTT |
|  | TXS | x |  | TGAAYAGAYGAAGATYATGTTAT |
| 1.1 | DBTNBT | x |  | GAAGYTAGATTTYAYAYAGTTTTGAAG |
|  | DBTNBT |  | x | ATCTTCRTTCACCACTATATT |
|  | DBTNBT | x |  | GAAGYTAGATTTYAYAYAGTTTTGAAG |
| 1.2 | DBTNBT | x |  | ATAYTTYAYAAAGTGGGTATTGT |
|  | DBTNBT |  | x | CARAACATATRCRTAARTRTCTCAATA |
|  | DBTNBT | x |  | TAAAYTYTAGGTGGTYYATYAA |
| 2.1 | DBTNBT | x |  | AAAYTYAATYGTAATAAYTTTTTAA |
|  | DBTNBT |  | x | ACTRRATCAAARATRAAACRAT |
|  | DBTNBT | x |  | TATAAAATAYAATGGAATGAGG |
| 2.2 | DBTNBT | x |  | TAAAATAYAATGGAATGAGG |
|  | DBTNBT |  | x | ARRCARTCRARAATATTTCCTCTTACCRC |
|  | DBTNBT | x |  | GAGGATTTTATTYTYGATTY |
