# Supplementary material for: The Epigenetic Regulation in Plant Specialized Metabolism: DNA Methylation Limits Paclitaxel in vitro Biotechnological Production
Source: Front Plant Sci. 2022 Jul 8;13:899444. doi: 10.3389/fpls.2022.899444 (PMC9305382; doi:10.3389/fpls.2022.899444)
Supplement: Supplementary file 2 [file Table_2.DOCX]

| *Supplementary material 2.* ***Cis*-acting regulatory elements involved in the defense and stress responsiveness present in promoter sequences.** | | | | | |
| --- | --- | --- | --- | --- | --- |
| ***Cis-*acting elements** | **Function** | **Sequence** | **Promoter** | | |
|  |  |  | **GGPPS** | **TXS** | **DBTNBT** |
| CGTCA-motif | cis-acting regulatory element involved in the **MeJA-responsiveness** | CGTCA |  | **×** |  |
| E-box | cis-acting regulatory element involved in the **MeJA-responsiveness** | CANNTG | **×** | x | x |
| G-box | cis-acting regulatory element involved in multi-function including **biotic/abiotic stresses and hormones-responsiveness** | CACGTG |  | **×** | **×** |
| TC-rich repeats | cis-acting element involved in **defense and stress responsiveness** | ATTTTCTTCA, ATTCTCTAAC |  |  | **×** |
| TGACG-motif | cis-acting element involved in the **MeJA-responsiveness** | TGACG |  | **×** |  |
